# A One-Square-Millimeter Compact Hollow Structure for Microfluidic Pumping on an All-Glass Chip

**DOI:** 10.3390/mi7040063

**Published:** 2016-04-09

**Authors:** Xing Yue (Larry) Peng

**Affiliations:** Department of Biology, School of Life Sciences, Xiamen University, Xiamen 361005, China; xypeng@xmu.edu.cn; Tel.: +86-592-288-0018; Fax: +86-592-288-0028

**Keywords:** lab-on-a-chip, all-glass, microfluidic, on-chip valve and pump, surface tension, glass microchip

## Abstract

A micro surface tension pump is a new type of low-cost, built-in, all-glass, microfluidic pump on a glass microchip fabricated by one-step glass etching. However, geometric minimization and optimization for practical use are challenging. Here, we report a one-square-millimeter, built-in, all-glass pump controlled by two-way digital gas pressure. The pump consists simply of two joint chambers and a piston between two gas control channels. It does not require pre-perfusion for initialization, and can immediately begin to run when a liquid enters its inlet channel. It is also more reliable than conventional micro pumps for practical use due to its ability to restart after the formation of a blocking bubble, which can serve as a valuable troubleshooting procedure. Its volumetric pump output was 0.5–0.7 nL·s^−1^ under a pump head pressure of 300 Pa.

## 1. Introduction

Currently, there are a lot of choices for a microfluidic system driven by micro pumps. We can have a valving membrane magnetically actuated by permanent magnets [1]; a polydimethylsiloxane (PDMS) diaphragm deformed by pneumatic pressure [2,3]; an electro-osmotic pump for flow injection [4]; a glass capillary with a mixture of PDMS–carbon nanopowder, silicone, and mineral oil serving as a piston [5]; a PDMS membrane with a piezoelectric actuator [6]; a flexible ultra-thin glass sheet deformed by pins [7]; a PDMS membrane with an electroplated thin-film permalloy strip for actuation [8]; a sandwiched polymer membrane deformed by voltages [9]; and an electro-statically actuated parylene membrane as a valve [10]. However, current micropumps were mostly designed involving multilayered structures [11], mostly moving parts or polymer membranes, which require complicated fabrication and packaging. More importantly, when we need a real built-in pump in a rigid material such as glass, the best optical material, we feel very difficult to find a pump that is easy to be integrated in the rigid chip by simple procedurals.

The rigidness of glass is a significant challenge for total-glass micropump integration. Using ultra-thin glass sheet, which can be bent and deformed to some extent, a total-glass pump was successfully realized by four serial valves sealing a cavity with two penetrating holes using flexible ultra-thin glass sheet [7]. Apart from this success, the glass’ poor flexibility limited the minimization of a bending-glass-sheet valve and, therefore, limited the minimization of the four-valve pump. More importantly, the two holes’ pins overlapping in the vertical dimension not only blocked the light along the vertical dimension, but also complicated the procedure of glass chip fabrication and packaging. Before we find any high-flexibility thin glass sheet and its supporting equipment for glass bending in the future, minimization of a totally transparent glass micropump is still uncertain.

If abandoning the fragile membrane, at least when there is no ideal glass membrane, we may go back to the micro surface tension devices [12,13], because this kind of device can be integrated on the two-dimensional plane without any vertical structure and only one-step glass etching is good enough and ready for glass bonding. Without any moving parts, with only a single geometric hollow space beneath the cover glass, it is transparent and lets the controlling gas enter from the side, with no opaque part or accessory destroying the glass’s optical nature. The disadvantage generated from the liquid-gas interface is mainly evaporation. In addition, the minimization and simplification of this kind of pump should be explored. This paper aims at the minimization and simplification of this kind of pump.

One-step glass etching is a simple, low-cost, and fast fabrication method for a series of essential monolayer microfluidic devices, performed on one microchannel monolayer at a time, including the micro surface tension alveolus (MISTA), pressure sensor (MISEN), piston (MISTON), valve (MISVA), and pump (MISPU). Controlled by digital gas pressure commands and constructed from only transparent glass without any moving parts, this all-glass microchip can be expected to evolve into a highly-integrated microfluidic chip or an integrated fluidic circuit (IFC) microchip. Thanks to digital gas-pressure control and a geometric structure composed of pure glass, the local redox reactions commonly caused by electrical controls that disturb the chemical environment inside the channels are not possible. Since micro- and nanofluidic devices, such as electro kinetic pumps, rely primarily on electricity and dissimilar materials, it is very difficult and cost-intensive to develop an ideal integrated microchip whose channel chemical environment is not disturbed by electricity and dissimilar materials [14]. Therefore, important aims are to minimize the size of the chip and to integrate microfluidic devices with similar materials and without the use of electricity, although fabricating an all-glass microchip that is not controlled by direct electricity is challenging. A nanofluidic electro-kinetic pump about the size of a red blood cell [15] and made from the same monolithic glass substrate has shown a promising future in the field of all-glass microfluidic devices, even though the electro kinetic principle does not eliminate the possibility of electrical interference in the chemical systems of nano- and micro channels. Furthermore, there are still high costs associated with pulsed-laser micromachining methods. In this paper, we report how to use one-step, wet etching of glass to fabricate a built-in micro pump with a one-square-millimeter area for practical use. The design of this novel micro pump is based on the theory of MISPU [13] and MISTA [12] and greatly improves upon its previous mother design.

## 2. Materials and Methods

### 2.1. Microchip Designs

The MISTA acted as the joints between channels. It is a key element in making the structure a functional pump. The pump consisted of five channels and two chambers connected by seven MISTA. For one-step etching fabrication, we need to calculate the isotopic etching speed for all channels, chambers, and MISTAs. To obtain a pump of 30 μm glass etching depth, we designed a 40 μm gap between two channels for a MISTA formation (one channel’s sharp end was pointing at the other channel). The photolithographic and wet chemical etching process eventually expands the design to make the two channels touch to form a MISTA for a MISEN, MISVA, MISTON, or MISPU. All structures of the pump were designed into a one mm square. A zigzag channel, not a straight channel, was designed to serve as a piston for space saving.

### 2.2. Microchip Fabrication

Glass microchips (Figure S1 in Supplementary Material) were fabricated by photolithographic and wet chemical etching techniques and thermo-compression bonding. The micro structure was designed for a lithography mask and applied on a 1.6 mm thick, 60 mm square glass substrate by a photolithographic procedure (glass substrate with chromium and S-1805 photoresist obtained from Shaoguang Microelectronics Corp., Changsha, China). The etching speed (HF:NH_4_F:HNO_3_ = 1:0.5:0.5 in molal concentration) was around 2 μm/min and the precise etching depth was controlled by inspection under a microscope. The glass bonding temperature was 560 °C [13].

### 2.3. Digital Gas Pressure Control

A N_2_ gas cylinder with a pressure regulator provided N_2_ gas pressure of 20–40 kPa. The pressure was transferred (Anthone Elec. Ltd., Xiamen, China) to digital pressures (e.g., 4 kPa) for pump driving. These digital pressure outputs were controlled by a computer program (software wrote by ourselves). The software set the pump cycle and sent the command sequences via a PC COM port to control the digital gas pressure applied on the outlet of the pump to drive the pump.

### 2.4. Optical Setup

The glass microchip was placed on an inverted microscope. The images were captured by a CCD camera. The experiments were also inspected by a metallurgical microscope (objective, coaxial LED white light source through the same objective) with a CCD camera over the microchip and a SONY video camera beside the microchip (see Figure S2).

### 2.5. Image Processing and Video Processing

The images and videos from the cameras or CCD, the pump cycle phase controlled by the software, and other information, such as time, were all synchronously showed on the computer’s screen, recorded to video clips by a screen recorder (Video S1–S9 in Supplementary Material).

### 2.6. Data Extraction and Pump Output Measurements

In the long-term pump output test, the water from the MISTA on a microchip was directed into a plastic tube (0.7 mm in diameter). The filling length was measured to determine the water volume for the pump output calculation. When the tube is set upright, this length represents the height of the water column, which was also under static pressure (Video S6 and Figure S3 in Supplementary Material).

## 3. Results and Discussion

### 3.1. Pump Principle

**Figure 1:**
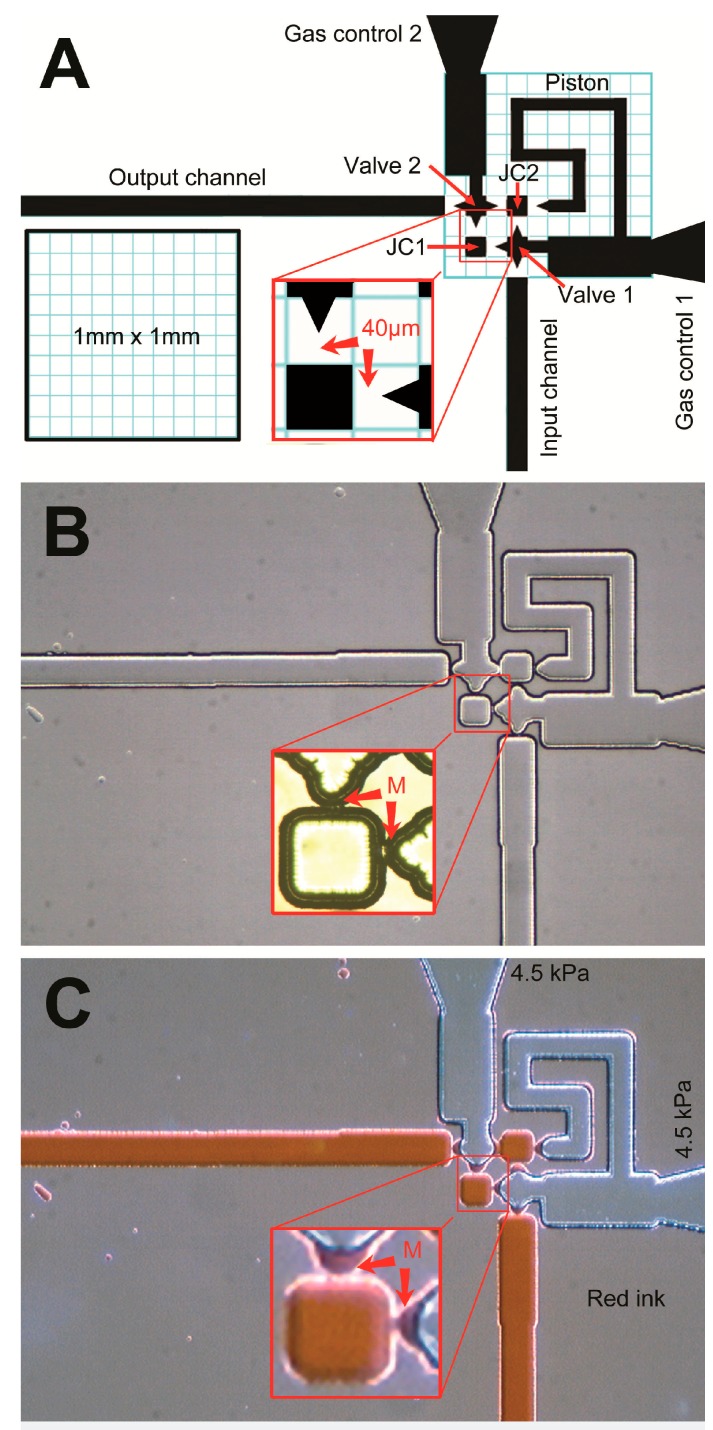
An actual design of a one-square millimeter MISPU and its fabrication by one-step glass etching. The black area of the photomask design (**A**) represents the etching area. The small **blue** grids in the one mm squares mark the size of 100 µm. Channels, chambers and valves separated by 40 µm gaps unite after 30-µm glass etching (**B**). The narrow connections help the high gas pressure (4.5 kPa) to form stable gas-liquid interfaces (MISTA, labeled by the red M) to stop microfluidic flow indicated in red ink (**C**). JC1: Joint chamber 1; JC2: Joint chamber 2.

The micro surface tension pump (MISPU) [13] is central to all micro surface tension devices. The pump is 4.2 mm long and 2.4 mm wide (about 10 mm^2^). With two MISVAs and one MISTON, the pump outputs liquid into a 300-µm-wide microchannel at a speed of 10 nL·s^−1^. To develop the pump for practical use, we sought to improve it in three ways: first, compressing the size of the pump channel system; second, reducing the three-way control to a two-way control; and third, enhancing its fault tolerance and recoverability. The strategy was to conserve the channel width for the pump output, fold the piston to save space, remove the loop channel for the valve, design a joint chamber to connect the two valves and the piston, and combine the input valve with the piston using a single-gas pressure control. Figure 1A shows an actual design of the pump. The piston channel is designed with a width of 60 µm. This piston channel measures 120 µm in width after 30-µm glass etching. The volume of the piston is about 7 nL. When the pumping cycle is set to 10 s, the pump output is about 0.8 nL·s^−1^. The piston channel opens into a wide channel (gas control 200 µm in width, and 500 µm in length). This wide channel is designed to stop the movement driven by the capillary force in the narrower piston channel and quantitatively define and stabilize one cycle of the pump output. We combined the piston and the input valve (valve 1) into gas control 1 because we used a single pressure command to simultaneously manipulate both the input valve and the piston. Low pressure opens the input valve and loads the piston with liquid passing through the input valve; high pressure closes the input valve and drives the piston to push its load into the next valve. Since the piston channel is longer than the input valve, the gas-liquid interface finishes its stroke in the input valve much earlier than it does in the piston channel. Therefore, at low pressure, piston loading follows the opening of the input valve; at high pressure, piston driving follows the closing of the input valve. This scenario is the working principle of combined gas control 1. Using an input valve and a piston, the three-way control is trimmed to form a two-way control, creating a valve-piston-valve pump.

### 3.2. Micropump Characterization

This novel MISPU design (see Figure 1A) still has two valves, although the input valve (valve 1) shares one gas control channel (gas control 1) with the piston. Unlike our previous valve design [13], the new valve design has no loop channel. The loop channel of the previous design is a channel connecting the valve that ends at one side channel. The loop channel helps the liquid in the valve enter and exit quickly, allowing the valve to open or close. Without this loop channel and due to the dead end of the valve, the valve cannot quickly clear liquid from its inner space using high gas pressure. Still, the loop channel has two main disadvantages. First, the loop channel occupies a great deal of space. Second, the loop channel decreases the valve’s fault tolerance and recoverability because bubbles accidentally entering the loop channel can decrease the pump efficiency or even stop the pump. A bubble in the loop channel is difficult to clear and, therefore, recoverability is not achieved. To solve this problem, we substituted the loop channel with another combined design: joint chambers between the valves and the piston. There are two joint chambers, each a square with dimensions of 100 × 100 µm^2^. One chamber connects the ends of the two valves. The other chamber connects the sides of the two valves to the end of the piston. During pumping, these chambers are always filled with liquid and function as tubes for the liquid while blocking the gas. Therefore, with no loop channel, the liquid in the two valves does not encounter a dead end and, instead, runs freely in and out of the joint chamber during the opening and closing of the valve. Since the joint chamber is much shorter than the long loop channel (just a square chamber), any bubble in the joint chamber is easily cleared, and the pump recovers.

### 3.3. Operation Details and Observations

**Figure 2:**
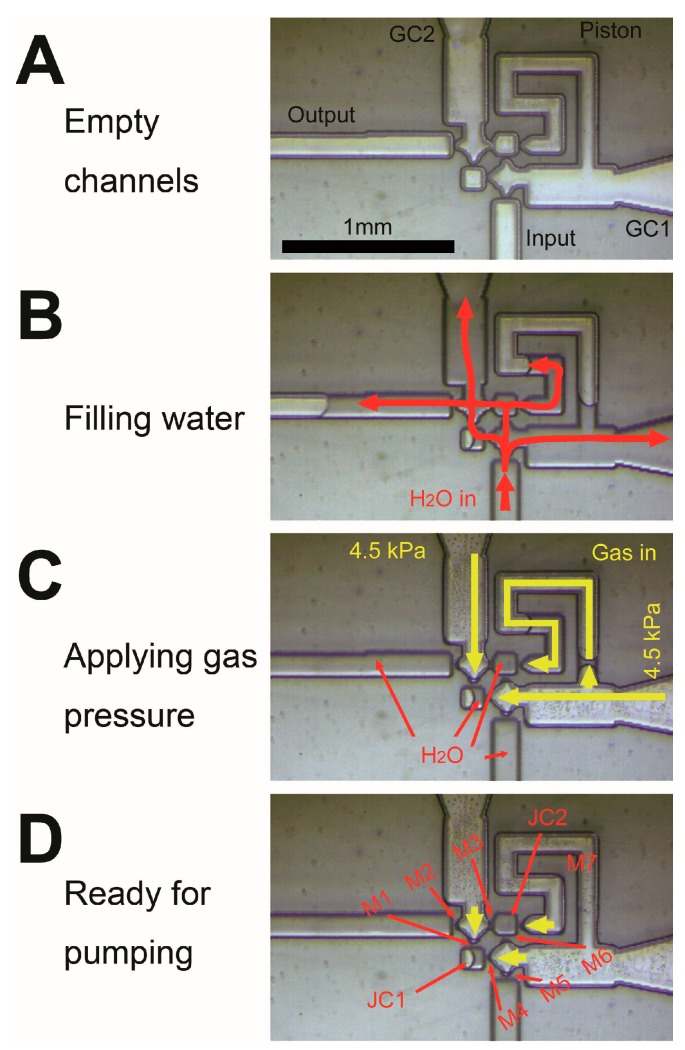
An initial preparing of a pump. The empty channels (**A**) are filled with water from the input channel ((**B**) see **red** arrows). When high gas pressure applied ((**C**) 4.5 kPa), water in control channels (GC1 and GC2) and in the piston are driven into liquid channels and the two joint chambers ((**D**) JC1 and JC2) until MISTAs (M1–M7) stop the liquid-gas interface. GC1: Gas control 1; GC1: Gas control 2.

Based on the photomask design (see Figure 1A), the etching depth was controlled at 25–30 µm during MISTA formation. These MISTAs eventually integrate the designs of all of the valves; the joint chambers and the piston form a microchannel system (see Figure 1B). These MISTA connections allow the passing of the liquid but do not allow the passing of the gas and prevent the gas from entering the liquid channels and the two joint chambers. The gas channels and liquid channels of the microchannel system under a high gas pressure of 4.5 kPa are shown in red ink in Figure 1C. The gas-liquid interfaces between the gas and liquid channels of the MISTAs are stable and clearly indicated in red (see inset), showing that the design and the one-step wet etching result in a stable microchannel system pump. Figure 1C also shows the pump’s initial ready state (see also Figure 2D). When high or low gas pressures are applied, the valves and piston are driven, and the pump begins to work. To clearly define its working principle and advantages over previous MISPUs, we needed to test the pump immediately. Figure 2 shows the initial preparation of the pump. Our previous MISPU pump design had loop channels. These loop channels require perfusion and must prevent any bubbles from entering at its start. Without the loop channels, the novel MISPU requires no special preparation to start pumping. The empty channels are inherently ready to pump liquid (Figure 2A). After conducting water through the input channel (Figure 2B), the liquid enters the channel system through the seven MISTAs (M1–M7) and two joint chambers (JC1 and JC2). When applying high gas pressure, the gas-liquid interfaces push the liquid in the gas channels back into the liquid channel, which stops at the seven MISTAs (Figure 2C,D), and the pump is ready to start pumping. The speed at which the liquid enters the pump through the input channel is controlled by the differential pressures of the input channel and the two gas control channels. Lowering the entrance speed of the liquid lowers the possibility that any bubbles remain in the channel system. Since there is no loop channel when conducting the liquid, the system is protected against bubbles. As shown in Figure 2B (see also the Supplementary Material movies), if perfusion occurs too quickly, some bubbles may remain in the piston channel because the liquid in M5 would have a shortcut through the open mouth of the piston channel and into the wide GC1 channel (Figure 2B,C). The bubble in the piston would be close to the open mouth such that when the high-pressure gas travels back into the GC1 channel, the bubble easily breaks and the piston channel can be easily cleared. Any bubbles in the two square joint chambers are also easy to clear because the MISTAs are very closely arranged, and differential pressure is easy to apply to the two chambers. Any bubbles in the output channel do not affect the pump’s performance, because the pump can push these bubbles forward (see the ESI movie). The simplest and fastest method to clear all of the bubbles in the channel system is to apply negative pressure to the output channel. This procedure causes the activity of the pump to resume, which demonstrates the pump’s robust recoverability (see the ESI movie). If a small bubble does not block the MISTAs, it does not affect the pump’s performance (e.g., a harmless bubble in JC1; Figure 2B–D). The pump can start regardless of the bubble.

### 3.4. Robotization and Functional Testing

**Figure 3:**
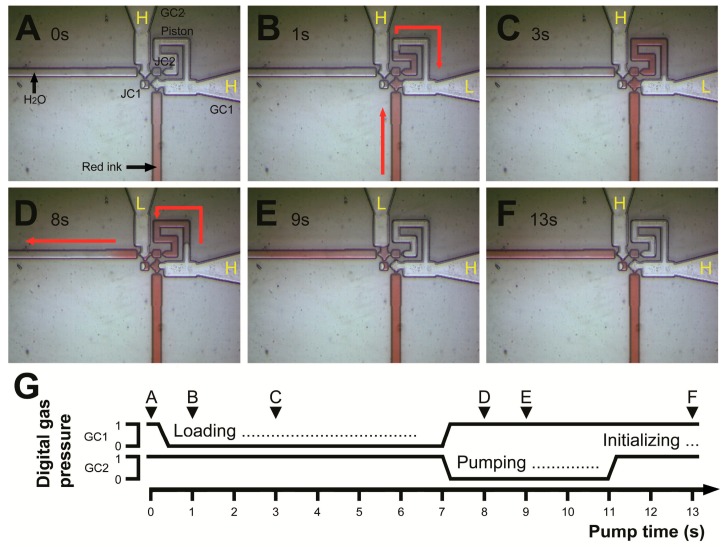
A 13 s pumping cycle controlled by two-way digital commands. After the pump is initialized ((**A**) 0 s), a pump cycle starts loading and red ink enters into the piston through JC2 ((**B**) 1 s), red arrows; ((**C**) 3 s). At 7 s, the pump starts pumping and red ink in the piston is pushed into the output channel ((**D**) 8 s), **red** arrows; ((**E**) 9 s). At 11 s, the pump is reset by a initializing process and ready for next pump cycle ((**F**) 13 s). The three basic processes (loading, pumping, and initializing) of a pump cycle are controlled by digital gas pressure. (**G**) 1 represents high pressure of 4.5 kPa and 0 represents low pressure of 2.8 kPa applied on GC1 and GC2). GC1: Gas control 1; GC2: Gas control 2; H: High pressure; L: Low pressure; JC1: Joint chamber 1; JC2: Joint chamber 2.

**Figure 4:**
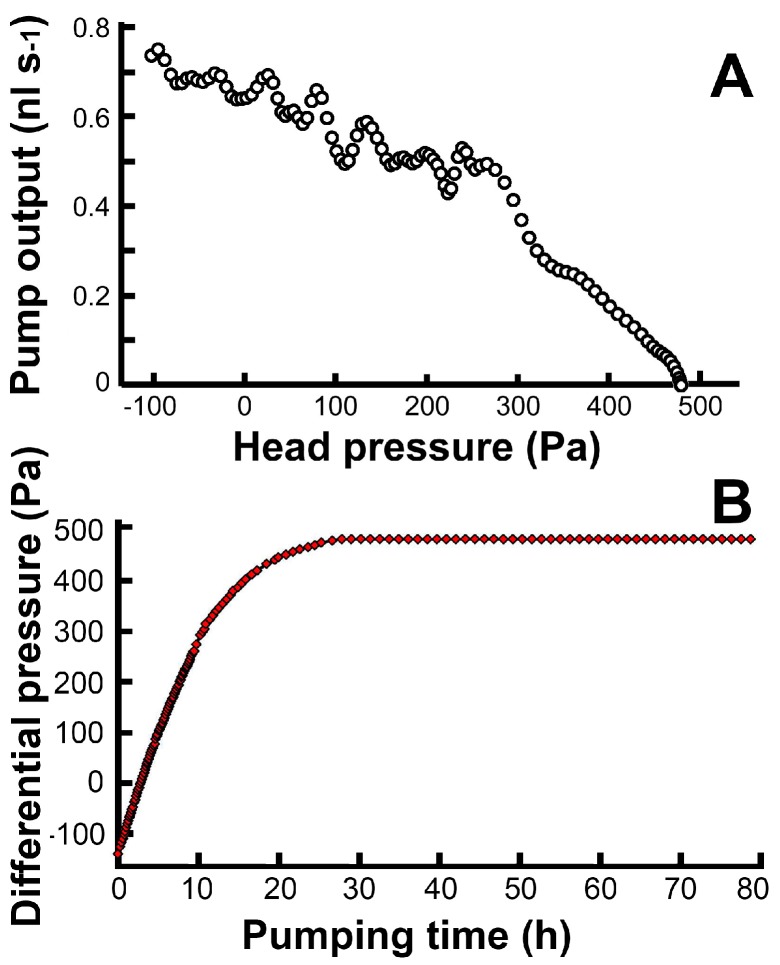
The relation between the volumetric pump output and the pump head pressure (**A**); the head pressure is the measured differential pressure (**B**) between the input channel and the output channel of the pump.

For the microchannel system of the pump, we programmed a very simple digital gas pressure command sequence (Figure 3). The initialized pump (Figure 3A) starts to operate when GC1 is switched from “1” to “0” (high pressure to low pressure). The low pressure in the GC1 channel opens valve 1 and conducts the liquid into the piston channel (Figure 3B), until the gas-liquid interface reaches the open area of GC1. This process occurs due to the decreased capillary effect in the wider channel (Figure 3C). The filling of the piston with liquid is the process of loading. After loading, GC2 is set to “0” to open valve 2, and GC1 is set to “1” to close valve 1 and push the piston (Figure 3D,E). The gas-liquid interfaces stop in front of the two joint chambers, finish pumping, and wait for the next process. When GC2 is set back to “1” to close valve 2, the pump is initialized for the next pumping cycle (Figure 3F). This command sequence continues to repeat, and the pump continues to run. The details of the entire cycle are shown in Figure 3G. While maintaining a cycle time of 10 s, the volumetric pump output was measured during a single continuous 30-h pumping session (Figure 4A). The pump’s head pressure is about 300 Pa, and its volumetric pump output is 0.5–0.7 nL·s^−1^. Longer testing of up to 80 h (Figure 4A) shows that the pumping is stable but loses its efficacy (Figure 4B) because of the backpressure (~470 Pa) from the output channel from 30–80 h.

### 3.5. The Working Principle of the Gas-Liquid Interface and Its Variants

The gas-liquid interface is actually a small hole serving as a block (or valve) for gas and a passage for liquid. Thus, the essential function of this interface is to block the gas when liquid goes through the small hole. The gas pressure will drive gas into the liquid to form a meniscoid bubble. If the gas pressure is too high, this meniscoid bubble will break into the liquid channel to fill the liquid channel with gas, causing valve failure. If we hope to close the valve very tight, preventing liquid leakage, we need to apply higher and higher pressure on the hole below the maximal pressure. Theoretically, the maximal pressure (*Pmax*) can be estimated by the Young-Laplace equation:

(1) $$Pmax=\gamma \left(\frac{1}{R1}+\;\frac{1}{R2}\right)$$

where γ is the surface tension, and *R*1 and *R*2 are the perpendicular or maximal and minimal principal radii of the curvature (mostly decided by the radii of the hole and close to the radii of the hole) when the hole is not a perfect circle. The higher the surface tension or the smaller the hole is, the higher the *Pmax* we can use (see Video S7).

In this study, we applied glass etching to form the small hole as gas-liquid interface. This was just a method of simple fabrication for the glass chip. Based on the principle of the interface, any material or method can be applied to fabricate a smaller hole. At 20 °C, the surface tension of pure water is 0.0728 N·m^−1^. If the radius of the hole is 20 μm, the *Pmax* is 7.2 kPa, similar to this study. If the radius of the hole is only 0.2 μm, no matter what fabrication methods on what material, the *Pmax* can be as high as 720 kPa. Thus, when we use this interface to control nanofluids with a hole of this size, it will make the pump’s gas-liquid interface more stable at high working pressure.

### 3.6. The Principle of Digital Pressure Setup

To set the digital pressure for the operation of the pump, we simply adjusted the high pressure (as 1 pressure) and low (as 0 pressure) according to the online image of what the microscopy monitor showed.

The method of how to set the high pressure was discussed before in Section 3.5. The high pressure was normally set close to the *Pmax* but not too close, in order to avoid the breakthrough of the bubble. For the low pressure, Video S8 showed that if the low pressure was too low (about 1–2 kPa), the inlet valve was overloaded and filled with liquid from the input channel. Even though this overloading did not suspend the pumping, it did make the pumping output difficult to quantify. Thus, the low pressure was adjusted to make the liquid stop at the gate of each channel (see Video S9), to make sure that each loading just filled the zigzag piston channel. In the process of adjusting the low pressure, we tended to choose a lower pressure (not too low, about 2.8 kPa) because lower pressure accelerated the loading process of piston channel.

### 3.7. The Relation between the Pump Output, Head Pressure, and Actuation Pressure

The relation and head pressure is shown in Figure 4A. The pump provides a head pressure of about 470 Pa. Excessive backpressure, which increases the leakage of the valve, reduces the pump output to zero (Figure 4B). At a proper low backpressure, the pump enters a state of normal operation (described in Section 3.6), exports a fixed volume from the piston channel for each single actuation, and its average pump output depends on the actuation frequency. Doubling the frequency means doubling the pump output, but the frequency cannot be too high because each actuation time cannot be too short. In the experiment of this design, one cycle of actuation could not be less than five seconds. The extension of the cycle time does not extend the functional actuation time, but extends the waiting time. Thus, the pump output is inversely proportional to the cycle time when the cycle time is over its minimal safe value.

Higher actuation pressure provides tighter valve function and shorter actuation time, but needs smaller holes to serve as valves. With the simple wet glass etching technique, the limitation of the size of the hole is about 20 μm, which means the maximal actuation pressure is about 5 kPa. Application of other techniques generating smaller holes (see Section 3.5) gives higher actuation pressure and the pumping frequency could go up to 0 Hz, 10 Hz, or even higher kHz, depending on how small the holes are made. Meanwhile, higher actuation pressure provides higher head pressure. So, a pump with smaller holes obviously expands its usage by its higher frequency and higher head pressure.

### 3.8. The Method of Pressure Control for Surface Tension Changing

In this case, pure water of known surface tension was the experimental liquid to pump. After adding red ink to label the liquid, the surface tension and the contact angle of the solution (liquid surface to the channel wall) changed a little but did not affect the state of the working pump (see Video S9). Different solutions of different surface tensions and different contact angles require adjustment on the high pressure and low pressure inputs (digital pressure setup, see Section 3.5 and Section 3.6). When pumping a surfactant solution, the high pressure should be lowered to avoid gas entering the liquid channel (see Section 3.5). A lower high pressure diminishes the head pressure, loosens the valve, and lengthens the actuation time. In the experiment of pumping a surfactant solution, we found that this low surface tension and high wettability surfactant solution (small contact angle) affected the pump efficiency. We also tried to pump culture medium (DMEM). Results showed that the pump could work with this kind of solution of glucose, protein, buffer, and other molecules. However, particles in the culture medium should be filtered, because particles or debris could block the small holes of the pump. Apart from that, the channel wall’s surface could be modified by absorbing molecules in the medium, and this modification might influence the pump’s state. For the promotion of this new technology, the stabilization of the surface tension and the contact angle (affect the capillary pressure) is the first thing to be taken into account. If not, we can use relatively clean solution similar to pure water to pump.

### 3.9. Possible Future Works

The pump did not have a vertical dimensional structure, not because it could not have, but because a flat pump could be easily integrated by one-step glass etching. Thus, the integration of this kind of pump and the three-dimensional or multilayer designs could be a future work. Considering that the evaporation on the interface of water and gas might unacceptably reduce the liquid volume, it is useful to find a proper material [6] which could be put at the interface to seal and prevent the evaporation. In addition, the structure of the pump comprised of only two dimensional geometric hollow channels and chambers; its polymer version could be very easy and cheaply fabricated by hot embossing [16].

## 4. Conclusions

A micro surface tension pump (MISPU) for an all-glass microfluidic chip has been optimized. One zigzag piston and two valves are integrated into a one-square-millimeter device using two square joint chambers. Controlled by a two-way digital gas pressure command sequence, the pump outputs liquid at a speed of 0.5–0.7 nL·s^−1^ and a head pressure of about 300 Pa. The pump’s outstanding fault tolerance and recoverability provide a long-term on-chip method to prevent bubble blocking.

## Supplementary Materials

The following are available online at http://www.mdpi.com/2072-666X/7/4/63/s1: Figure S1: A one-square-millimeter, built-in, all-glass, active microfluidic pump. The pump controlled by digital gas pressure (gas control 1 and gas control 2) pushes liquid from the input channel into the output channel. Figure S2: The pump test platform. The digital gas pressure command sequence is conducted to the microchip (on a microscopic platform) using two plastic tubes (white tube for gas control 1 and black tube for gas control 2). Water from a bottle (fixed water level of 26.5 mm’s high, see video 6) is conducted to the input channel of the microchip. The pump output is conducted to a plastic tube upright beside a ruler. The water level in the output tube is labelled by red ink and recorded by a video camera for post pump output measurements. Figure S3: Data collected in the experiments shown in Figure S2. The pump’s head pressure and its volumetric pump output in Figure 4 is calculated by the differential output level with time. Video S1: The water from the input channel is entering the empty pump. Video S2: The pump is initiallized by setting the two gas control channel to “1”. “1” represents the high digital level of gas pressure. Video S3: The pressure effect of “0” on pump’s working state. Video S4: The recover of the pump by clearing the channels using negative pressure. Video S5: The mechanism of pumping is indicated by red ink. Video S6: The recording of the output level in the tube (left) and the pump state (right) during the 30-h test. Video S7: The liquid-gas differenttial pressure according to the shape of the liquid-gas interface. This interface is a hole-like joint in the pump controlled by gas pressure. Data showed in this video [12] tells us that the highest pressure can be set to 4.5 kPa. Video S8: The inlet valve gas channel (GC1) is filled with liquid (overloaded) from the input channel when the low digital pressure is too low (about 1–2 kPa). Video S9: The inlet valve gas channel (GC1) overloading is avoided when the low digital pressure was proper (about 2.8 kPa).
