# Supplementary material for: A One-Square-Millimeter Compact Hollow Structure for Microfluidic Pumping on an All-Glass Chip
Source: Micromachines (Basel). 2016 Apr 9;7(4):63. doi: 10.3390/mi7040063 (PMC6190336; doi:10.3390/mi7040063)
Supplement: Supplementary file 1 [file micromachines-07-00063-s001.pdf]

# Supplementary Materials: A One-Square-Millimeter Compact Hollow Structure for Microfluidic Pumping on an All-Glass Chip

**Xing Yue (Larry) Peng**

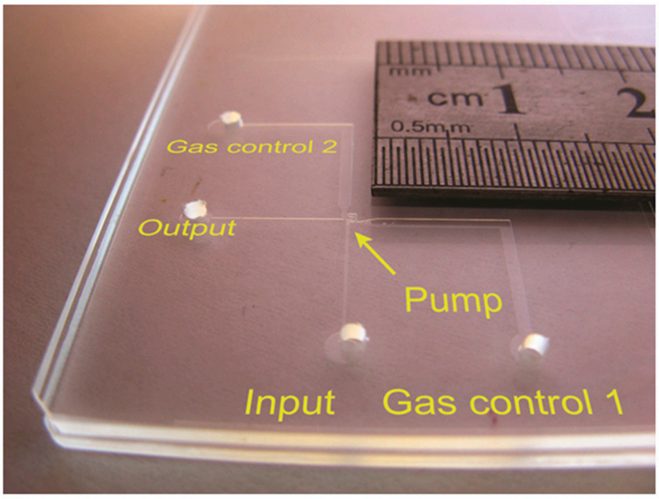


**Figure S1.** A one-square-millimeter, built-in, all-glass, active microfluidic pump. The pump controlled by digital gas pressure (gas control 1 and gas control 2) pushes liquid from the input channel into the output channel.

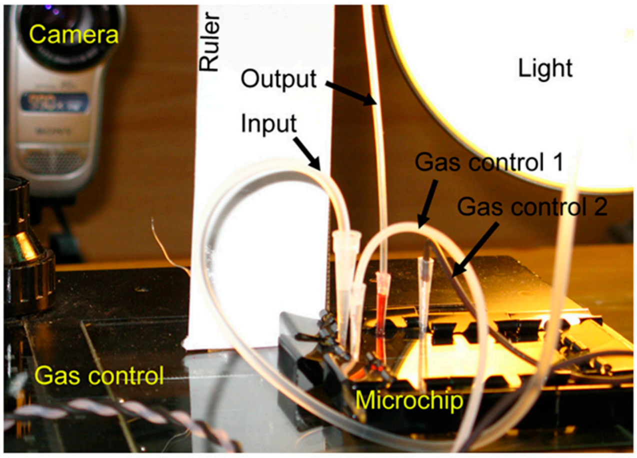


**Figure S2.** The pump test platform. The digital gas pressure command sequence is conducted to the microchip (on a microscopic platform) using two plastic tubes (white tube for gas control 1 and black tube for gas control 2). Water from a bottle (fixed water level of 26.5 mm's high, see Video S6) is conducted to the input channel of the microchip. The pump output is conducted to a plastic tube upright beside a ruler. The water level in the output tube is labelled by red ink and recorded by a video camera for post pump output measurements.

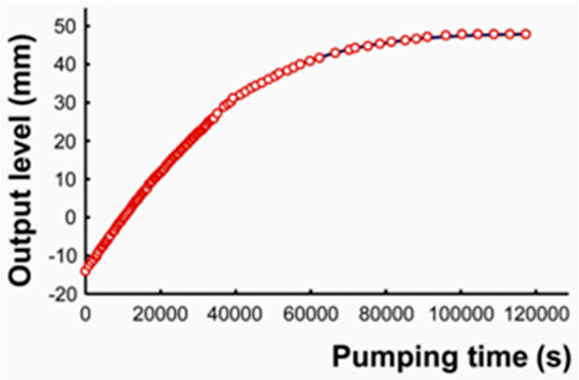


**Figure S3.** Data collected in the experiments shown in Figure S2. The pump's head pressure and its volumetric pump output in Figure 4 is calculated by the differential output level with time.
